# A simulation study evaluating how population survival and genetic diversity in a newly established population can be affected by propagule size, extinction rates, and initial heterozygosity

**DOI:** 10.7717/peerj.16628

**Published:** 2024-01-15

**Authors:** Iván Vera-Escalona, Antonio Brante

**Affiliations:** 1 Departamento de Ecología, Facultad de Ciencias, Universidad Católica de la Santísima Concepción, Concepción, BioBío, Chile; 2 Centro de Investigación en Biodiversidad y Ambientes Sustentables (CIBAS), Universidad Católica de la Santísima Concepción, Concepción, BioBío, Chile

**Keywords:** Forward-in-time, Simulations, Invasive species, Non-native species, Marine species

## Abstract

The introduction and establishment of invasive species in regions outside their native range, is one of the major threats for the conservation of ecosystems, affecting native organisms and the habitat where they live in, causing substantial biological and monetary losses worldwide. Due to the impact of invasive species, it is important to understand what makes some species more invasive than others. Here, by simulating populations using a forward-in-time approach combining ecological and single polymorphic nucleotides (SNPs) we evaluated the relation between propagule size (number of individuals = 2, 10, 100, and 1,000), extinction rate (with values 2%, 5%, 10%, and 20%), and initial heterozygosity (0.1, 0.3, and 0.5) on the population survival and maintenance of the heterozygosity of a simulated invasive crab species over 30 generations assuming a single introduction. Our results revealed that simulated invasive populations with initial propagule sizes of 2–1,000 individuals experiencing a high extinction rate (10–20% per generation) were able to maintain over 50% of their initial heterozygosity during the first generations and that under scenarios with lower extinction rates invasive populations with initial propagule sizes of 10–1,000 individuals can survive up to 30 generations and maintain 60–100% of their initial heterozygosity. Our results can help other researchers better understand, how species with small propagule sizes and low heterozygosities can become successful invaders.

## Introduction

Invasive species, are a small group of non-native species introduced by humans in areas outside their native range where they can establish (Lodge et al., 2006), reproduce, and generate significant losses for the environments of invaded regions of the world (Diagne et al., 2020; Heringer et al., 2021). These species can affect native organisms by competing, preying on, displacing, hybridizing, excluding, and even extinguishing them (Mooney & Cleland, 2001; Gurevitch & Padilla, 2004; Williams & Grosholz, 2008). Invasive species can seriously affect ecosystems, the biodiversity, and the economy of countries more exposed to invasions (Manchester & Bullock, 2000), causing millions of dollars loses due to environmental damages, native species impacts, and human diseases (Pimentel, Zuniga & Morrison, 2005; Hoffmann & Broadhurst, 2016).

The study of biological invasions has been a concern for researchers from different fields who have tried to elucidate how invasions occur and their potential consequences (Mooney & Hobbs, 2000; Leppäkoski & Olenin, 2000; Pagad et al., 2018; Prior et al., 2018; Pyšek et al., 2020). Thus, the evaluation of data and models assessing the risk of a species to become invasive in the future, framed in the so-called Population viability analyses (Boyce, 1992), have studied the relative importance of different ecological, physiological, and evolutionary aspects of invasive species that explain why some species become invasive. Researchers have found that the successful introduction and establishment of invasive species relies on the combined effects of life histories of species, environmental tolerance, reproductive suitability, habitat suitability, propagules size (number of individuals of a species that is introduced in a new environment during one event), propagule pressure (the composite measure of propagule size and number of invasions), and spatiotemporal characteristics of propagule arrival (Davis, 2009; Simberloff, 2009). For instance, a recent simulation-based study analyzing what factors contribute the most to the success of an invasive species have found that propagule size, propagule pressure and risk-release curve are the most important factors to determine the potential success of the establishment of non-native species in a new area (Stringham & Lockwood, 2021). Besides strictly ecological and demographic aspects of invasive species, studies have documented that the success of non-native species to become invasive might also depend on the genetic diversity of the propagule because propagules with low genetic diversity might result in the loss of genetic variability and inbreeding depression due to bottlenecks, causing a non-successful invasion (Suarez & Tsutsui, 2008; Fitzpatrick et al., 2012). Nevertheless, some studies have revealed that invasive species with large propagule sizes and high propagule pressure can overcome the effects of extinction rate (probability of an occurring event per generation leading to the extinction of the population) and bottlenecks on their genetic diversity while other studies have suggested that a reduction of genetic diversity might have little effect on invasion success even in single events with small propagule size (Frankham, 2005; Davis, 2009; Kaňuch, Berggren & Cassel-Lundhagen, 2021). Empirical studies focused on the role of propagule size and extinction rate over changes on the maintenance of genetic diversity and population survival could potentially help explaining why some non-native species are more prone to succeed as invaders in new regions than others. Nevertheless, cases where information of introduced species is known in real time and tracked through time, including changes in demographic, ecological, and genetic traits are uncommon. However, our understanding of the effects of these traits can be informed using simulated data

Niche and ecological simulation-based studies have been used during the last decades to understand how certain landscape and biological traits can explain the presence of species, evaluating the likelihood of this species to survive under future conditions (Benazzo et al., 2015). In this way, simulation-based approaches, through the evaluation of ecological models, could help predict what species could potentially be more invasive than other to reach and establish into certain areas of the world, and ultimately become invaders in these regions (Drake & Lodge, 2004; Guisan et al., 2014; Tisseuil et al., 2016). Along with ecological models and niche models, genetic simulations could help to predict what makes species more successful to concrete an invasion and thus can help to determine what species could potentially be more invasive than others based on their genetic diversity (Jeschke & Strayer, 2008; Jiménez-Valverde et al., 2011; Ferrari, Preisser & Fitzpatrick, 2014; Srivastava, Lafond & Griess, 2019). Predictions of the genetic diversity of potential invasive species can be estimated through forward-in-time simulations. Forward-in-time simulations are ecological-genetic simulations based on individuals that can be created from real data (*i.e.,* genetic and ecological data collected from species), simulated data (*i.e.,* genetic and ecological data created under idealized conditions) or both, where every single simulated individual follows a life cycle (*e.g.*, simulated individuals born, grow, reproduce and die) allowing to monitor changes in the demographic and genetic composition of populations at specific time intervals (Carvajal-Rodríguez, 2010; Hoban, Bertorelle & Gaggiotti, 2012). So far, forward-in-time simulations have been used in conservation studies to evaluate how species will respond to different changes in the landscape including those linked to climate change (Grossen et al., 2020) and landscape modifications (Vera-Escalona et al., 2018). Nevertheless, to our knowledge, no simulated study has combined yet the ecological and genetic attributes of species under a forward-in-time approach to predict the invasiveness degree of non-native species and changes in the maintenance of the genetic diversity (*i.e.,* heterozygosity) through time as consequence of different ecological, demographic, and genetic scenarios.

Previous studies have focused on the number of individuals and habitat suitability that make an invasion successful (*e.g.*, Britton & Gozlan, 2013; Blackburn, Lockwood & Cassey, 2015; Duncan, 2016; Moulton & Cropper, 2019; Saccaggi, Wilson & Terblanche, 2021), as well as on the effects of propagule pressure and invasibility (Davis, 2009), but less attention has been paid to the combined effect of propagule size, extinction rate and initial genetic diversity on population survival and the maintenance of the genetic diversity from source populations. With this in mind, we evaluate the effects on the survival and heterozygosity of a population with different propagule sizes, extinction rates, and initial heterozygosities using a stochastic model. For this purpose, we simulated a crab species, since invasive crabs are a well-studied group of invaders and have been described as a major force of change in coastal ecosystems, producing a high loss of native organisms, affecting even the economy of coastal regions (Griffen & Byers, 2009; Howard, Therriault & Côté, 2017). Crabs are a potential threat not only for regions highly inhabited by humans, but also in more isolated areas like Antarctic peninsula, where crabs have been described as potential invaders where they can arrive through ballast waters and fouling from maritime transports (Mooney & Hobbs, 2000; Ruiz et al., 2000; Fofonoff et al., 2003; Firestone & Corbett, 2005; Griffiths et al., 2013; Aronson et al., 2015).

## Materials and Methods

**Figure 1:**
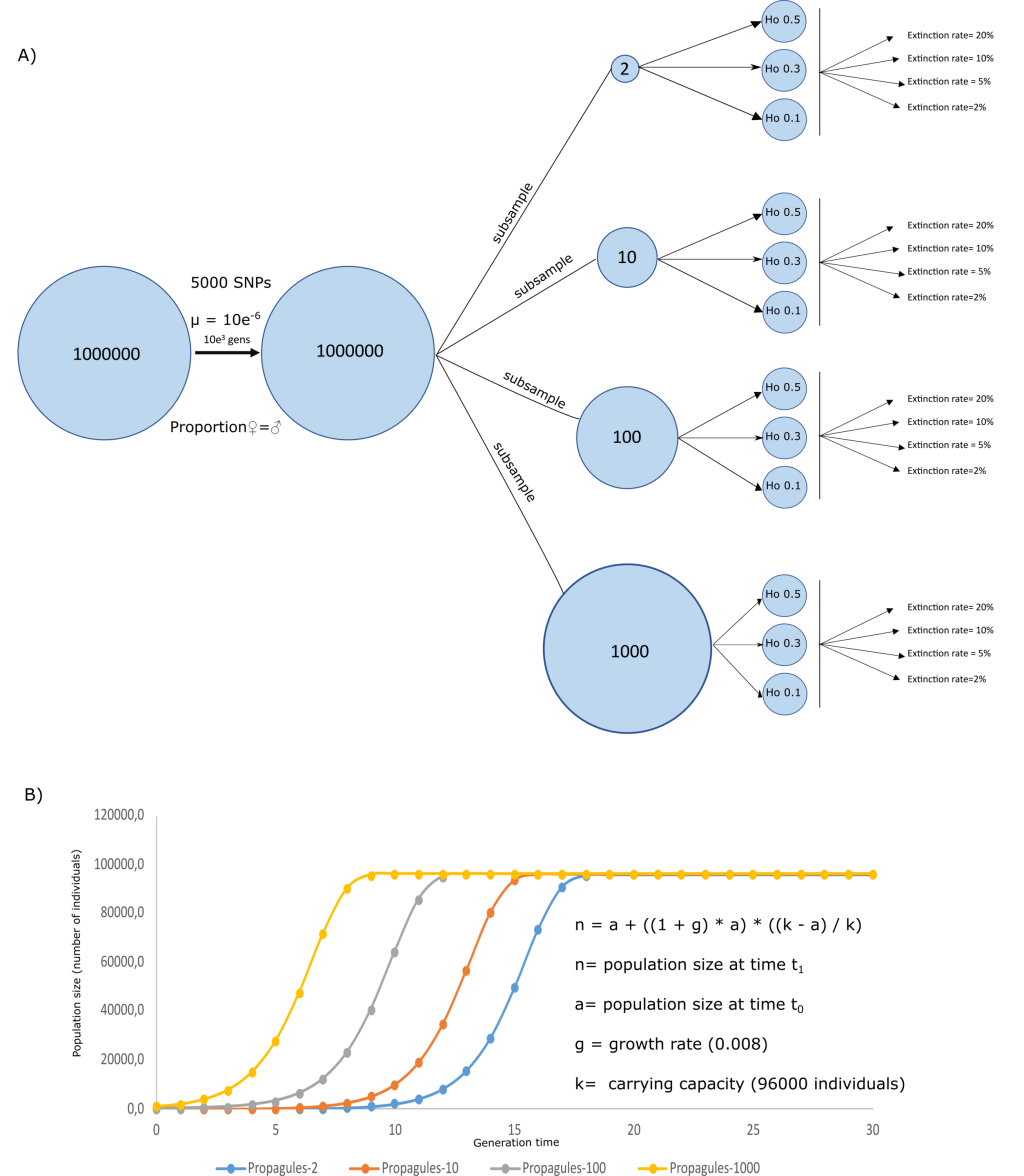
Simulation schemes used. Simulation schemes used to evaluate the effects of propagule size (*n* = 2, 10, 100, and 1,000) with different initial heterozygosities (0.5, 0.3, and 0.1) and extinction rate (2%, 5%, 10%, and 20%) from a simulated invasive crab species using a forward-in-time approach (A). Initial conditions for the logistic growth with a maximum carrying capacity = 960,000 individuals, assuming an area of 1,000 m^2^ using a simplified formula for logistic growth for our models assuming a stochastic effect of extinction rate according to the algorithm used in Nemo 2.3.56 (B).

A simulated crab species, a species created through simulating ecological and genetic attributes of a real crab species, was created in Nemo 2.3.54 (Guillaume & Rougemont, 2006) to evaluate the effects of initial heterozygosity, propagule number, and extinction rates on the survival and genetic diversity of a population. Genetically, the simulation assumed a population size = 1000,000 individuals exhibiting Hardy Weinberg equilibrium, random mating, equal sex ratios, individual fecundity of 100, 5,000 biallelic neutral SNPs, and a mutation rate of 5e−6. The simulations ran for 1,000 generations, by when the population has likely become well established and genetically stable. After 1,000 generations, four subsamples were taken into new analyses, to set the initial conditions of the propagule from the scenario schemes from Fig. 1. Using these subsamples, we were able to simulate four propagule sizes, with *n* = 2, 10, 100, and 1,000 (Propagules-2, Propagules-10, Propagules-100, and Propagules-1000, respectively) introduced in a new region during a single-event. Each subsample was then simulated under stable demographic conditions during 500 generations until reaching three different heterozygosity scenarios Ho = 0.5, 0.3, and 0.1 (Fig. 1A). Extinction rates are crucial for estimating the risk of population extinction (Ripa & Lundberg, 2000) but might also be an important limitation and source of bias for the approach used here since extinction rates are mostly unknown from experiments and observations in nature. To reduce the bias in our simulations we used a wide range of possible extinction rates that could be flexible enough to interpret the results under different perspectives. Therefore, each propagule size and heterozygosity scheme were exposed to different extinction rates, assuming that all propagules arriving at a new area could face different levels of extinction, affecting the survival of the species differently. Extinction rates included in the analyses were 2%, 5%, 10%, and 20%.

Twenty replicates were used for each simulation. Demographic and genetic changes (*i.e.,* heterozygosity) in populations with different initial propagule size were evaluated during 30 generations following a logistic model where every population of the invasive crab could reach a carrying capacity = 96,000 (Fig. 1B). This way, simulated populations could reach a carrying capacity of 96 individuals per m^2^ in an area of 1,000 m^2^, corresponding to the area inhabited by a coastal species introduced in a coast of 2,000 m length and up to 5m below the lowest tide. These ecological traits of the simulated species were based studies of invasive crabs, including average populations from *Carcinus maenas* and *Hemigrapsus sanguineus* (Breteler, 1976; Kraemer et al., 2007; O’Connor, 2014). Therefore, the four simulated scenarios could simplify four crab propagule sizes, reaching their capacity at different times from the first introduction. To evaluate the success of the invasion, survival rate measured as number of replicates overcoming extinction (n number of survival replicates/20 replicates), along with heterozygosity (as calculated by Nemo) as a measure of genetic diversity, were calculated to observe the likeliness of species to survive and maintain its genetic pool from a source population. All simulated scenarios were uploaded to figshare (https://figshare.com/s/44af47055dc3b663cec9).

## Results

### Heterozygosity 0.1

**Figure 2:**
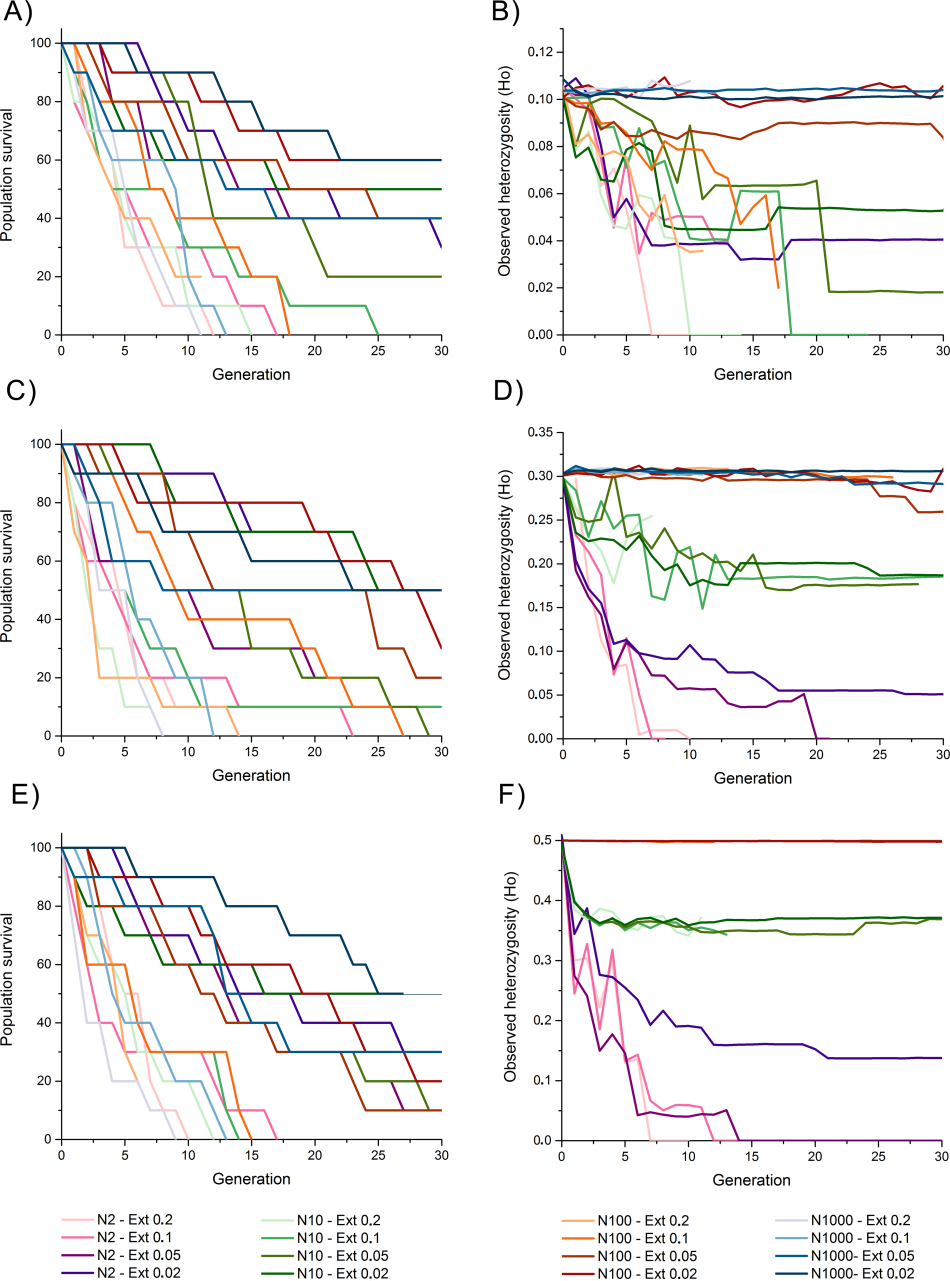
Survival rate (measured as the percentage of replicate surviving in each generation) and observed heterozygosity through generation time for simulations of an invasive crab-like species. Survival rate (measured as the percentage of replicate surviving in each generation) and observed heterozygosity through generation time for simulations of an invasive crab-like species with a propagule size = 2, 10, 100, and 1,000 individuals (Propagules-2, Propagules-10, Propagules-100, and Propagules-1000 respectively), extinction rates = 0.2 (20%), 0.1 (10%), 0.05 (5%), and 0.02 (2%), and initial heterozygosity = 0.5 (A–B), 0.3 (C–D), and 0.1 (E–F).

Survival rates among populations with initial heterozygosity = 0.1 revealed a trend where populations with higher extinction rates (*i.e.,* 10% and 20%) presented the lowest survival rates despite the propagule size, suggesting a higher effect of extinction rate over population size for survival rates, including total extinction by generation 11 to 25 (Fig. 2A). Populations with lower extinction rate (*i.e.,* 2% and 5%) revealed a higher survival rate of 60–90% by generation 10, and 20–60% survival rate by the end of the simulations. This survival was higher for populations with larger propagule sizes.

The maintenance of heterozygosity revealed that populations with larger propagule sizes (*i.e.,* 100–1,000 individuals) and low extinction rates (*i.e.,* 2% and 5%) maintained 85–100% of their initial levels of heterozygosity until the end of the simulations (Fig. 2B). In all other simulated scenarios, 20–45% of the initially heterozygosity was lost during the first five generations, and 25–100% of the initially heterozygosity was during the subsequent generations. Only populations with lower extinction rate that did not become extinct remained part of their initial heterozygosity by the end of the simulations.

### Heterozygosity 0.3

Survival rates among populations with initial heterozygosity 0.3 were slightly higher than for scenarios considering an initial heterozygosity 0.1 with all simulated populations experiencing an extinction rate of 20% becoming extinct between generation 9 and 14 and those with a higher extinction rate surviving, in average, for longer generations (Fig. 2C). Survivability by generation 30 was similar to simulated scenarios with heterozygosity 0.1.

The maintenance of heterozygosity was high among populations with initial propagules of 100–1,000 individuals despite the extinction rate simulated, maintaining 86–100% of their initial heterozygosity (Fig. 2D). Simulated populations with initial propagule sizes of 10 individuals revealed a slower loss of heterozygosity through time than when compared with simulated populations with initial heterozygosity 0.1, maintaining approximately 70% of their initial heterozygosity by generation 10 and 55–60% of their heterozygosity by the end of the simulations, except for the simulated population experiencing a 20% extinction rate that became extinct by generation 7. Simulated populations with initial propagule size of two individuals lost 67–70% of their initial heterozygosity by generation 5. Although three of these populations became extinct and lost most of their heterozygosity, simulated populations experiencing an extinction rate of 2% maintained an 18% of its initial heterozygosity by generation 30.

### Heterozygosity 0.5

Populations with initial heterozygosity 0.5 with extinction rates 20% and 10% revealed a rapid decline of survival rate from generation 1 up to generation 19 where all populations got extinct regardless the initial number of propagules (Fig. 2E). Survival rate declined at a slower rate under the scenarios considering extinction rates 5% and 2% (Fig. 2E). By generation 10, all populations under extinction rates of 5% and 2% presented a survival rate larger than 60%. Nevertheless, by the end of the simulations at generation 30, only two simulated scenarios with an extinction rate 2% presented a survival rate greater than 50%.

Populations with initial propagule sizes 100 and 1,000 individuals that remained alive by generation 30 maintained near 100% of the initial heterozygosity (Fig. 2F). Populations with initial propagule size = 10, maintained 75–80% of their initial heterozygosity by generation 30, while populations with initial propagule size = 2 rapidly lost 55–70% of their heterozygosity by generation 5. Populations with initial propagule size 2 suffered a rapid decline in heterozygosity and extinction but the populations experiencing an extinction rate 2% maintained 30% of its initial heterozygosity by generation 30.

## Discussion

The expansion of non-native species during the Anthropocene has been one of the most important threats for native species distribution and one of the major causes of the transformation of their habitats (Capinha et al., 2015; Darling & Carlton, 2018; McInerney, Doody & Davey, 2021). Here, by creating in-silico experiments using a forward-in-time approach we studied the ecological and genetic responses of a simulated invasive species. Our results suggest that extinction rate combined with a moderate to high number of propagule sizes explain why some non-native species survive, maintain a high genetic diversity, and become invasive after a single event of introduction. These results also suggest that the combined effect of low extinction rates and moderate to high propagule size can overcome the loss of heterozygosity through time. A brief comparison with information from the literature and potential implications from our results are discussed below.

### Survival rates under different scenarios

Introduced species can have different survivability likelihoods, especially due to lack of habitat, competition and other harsh conditions making them less likely to survive and suffer local extinctions (Carlton, 2000; Brown & Sax, 2004; Stohlgren & Schnase, 2006; Kulhanek, Ricciardi & Leung, 2011; Tingley et al., 2014; Jiménez-Valverde et al., 2011; Barney, Ho & Atwater, 2016; Geburzi & McCarthy, 2018). Population viability analyses mostly focus on propagule pressure and propagule size, and not always include the effect of survival rates and the relative importance of initial heterozygosity to maintain the genetic diversity that facilitate species to become invasives (Lockwood, Cassey & Blackburn, 2005; Cassey et al., 2018; Stringham & Lockwood, 2021). By creating in-silico experiments (experiments created with computer simulations) we observed that, under simulated conditions, populations from non-native species originated from a propagule size = 100–1,000 individuals experiencing an extinction rate lower than 5% can successfully establish outside their native range for at least 30 generations. Furthermore, we found that even an invasive population originated from a small propagule size with only 2–10 individuals can present a 30–50% survival during the first 5 generations of introduction under controlled conditions. These analyses have profound effects on the maintenance of the genetic diversity of likely invasive populations.

### Genetic diversity

Although the genetic composition of populations might play an important role in explaining how non-native species become invasive (Frankham, 2005; Kaňuch, Berggren & Cassel-Lundhagen, 2021), this is not always included in simulated models of biological invasions. The genetic diversity of propagules can be of interest for the study of non-native species, especially when introductions occur during multiple invasions or when invasions occur from large propagule sizes, which increases the genetic diversity and the likeliness of the success of the invasion (Lockwood, Cassey & Blackburn, 2005). Genetic studies have found that populations with large sizes tend to maintain the genetic diversity through time and this has been extrapolated for biological invasions, assuming that a successful biological invasion might be related to large propagule sizes maintaining the genetic diversity of invasive species, despite the number of introductions (Lande & Barrowclough, 1987; Frankham, 1996; Lockwood, Cassey & Blackburn, 2005; Memmott et al., 2005; Sinclair & Arnott, 2016). However, biological invasions can also occur from events of small propagule sizes and single events of introduction. Several mechanisms have been described to explain why some non-native species with small propagule sizes and single events of introduction can become successful invaders (*e.g.*, including self-fertilization and gene flow due to the increase of propagule pressure), maintaining a considerable amount of heterozygosity when invading a new region (*e.g.*, Frankham, 2005; Winkler et al., 2019).

Here, by using isolated populations and sexual reproduction among our simulated crabs, we controlled these mechanisms to focus only on the effects of propagule size, extinction rat, and initial heterozygosities on on the maintenance of the heterozygosity. Our results revealed that when extinction rate ranged between 2–5% per generation, a single event with an initial propagule size of *n* = 100–1,000 could be enough to maintain during 30 generations 100% of the initial heterozygosity when the initial heterozygosity of the propagules is 0.5 and maintain 86–100% of the initial heterozygosity when the heterozygosity of the propagule is 0.3. Moreover, even under a high extinction rate scenario (20% per generation), heterozygosity maintenance remained high in those populations. Although high heterozygosity values such as 0.3 and 0.5 could be considered high for a set of SNPs, this is not an atypical condition for invasive species (Frankham, 2005) and thus our results could also be representative of some species in the wild. Populations originated from events with smaller propagule sizes (*n* = 2–10) experimented the loss of nearly 50% of its original heterozygosity during the first 5 generations in all propagule heterozygosities simulated (0.1, 0.3, and 0.5) and exhibited lower survival rates. Although these results come from a simulated single event of introduction it might be extrapolated to multiple events of introduction, where an additive effect of the genetic diversity could occur, supporting studies warning the negative consequences of a high propagule pressure to maintain the genetic diversity of invasive species regardless of their low propagule sizes (Lockwood, Cassey & Blackburn, 2005; Simberloff, 2009). These results help better support previous studies, as occur with Kaňuch, Berggren & Cassel-Lundhagen (2021) that studied a terrestrial species (the bush-cricket *Metrioptera roeselli*), revealing that a small propagule size (2–32 individuals) can overcome the potential effects of bottleneck and even maintain or increase their genetic diversity after a small number of generations, generating the long-term persistence of an introduced species. Therefore, initial heterozygosity results essential for the success of the invasive species for maintaining the genetic diversity, especially when for populations with small propagule sizes.

## Conclusions

The success of a biological invasions depends on several factors including transport, habitat similarity, physiological tolerance, propagule pressure, propagule size, extinction rate and genetic diversity. Here, by using in-silico simulations with a forward-in-time approach we discovered populations with initial propagules of 10–1,000 individuals with initial heterozygosities 0.3 and 0.5 can maintain over 60% of their heterozygosity before extinction even under high extinction rates or up to 30 generations when extinction rates were low. Furthermore, populations with initial propagule size of 2 individuals and initial heterozygosities of 0.3 and 0.5 can maintain over 50% of their heterozygosity during the first 5 generations. Populations from propagule sizes of 100–1,000 individuals with initial heterozygosity 0.1 and low extinction rates per generation (2% and 5%) can maintain over 80% of their initial heterozygosity. Our results can help other researchers better understand how species with small propagule sizes and low heterozygosity can become successful invaders.

## Additional Information and Declarations

### Data Availability

The following information was supplied regarding data availability: The simulation files used in the article, including settings and simulated SNPs, are available at Figshare: Vera-Escalona, Iván; Brante, Antonio (2023). A simulation study evaluating how population survival and genetic diversity in a newly established population can be affected by propagule size, extinction rates, and initial heterozygosity. figshare. Dataset. https://doi.org/10.6084/m9.figshare.18957830.v1.
